# Incidence of venous thromboembolism and bleeding events in patients with lower extremity orthopedic surgery: a retrospective analysis of a Japanese healthcare database

**DOI:** 10.1186/s13018-017-0549-4

**Published:** 2017-04-04

**Authors:** Takeshi Fuji, Masao Akagi, Yasuyuki Abe, Eisei Oda, Daisuke Matsubayashi, Kaori Ota, Masafumi Kobayashi, Yasuyuki Matsushita, Jumpei Kaburagi, Kei Ibusuki, Atsushi Takita, Mikio Iwashita, Takuhiro Yamaguchi

**Affiliations:** 1 Department of Orthopaedic Surgery, Japan Community Healthcare Organization Osaka Hospital, 4-2-78 Fukushima, Fukushima-ku, Osaka 553-0003 Japan; 2 Department of Orthopaedic Surgery, Kinki University Faculty of Medicine, Osaka-Sayama City, Osaka Japan; 3 Department of Orthopaedic Surgery, Federation of National Public Service Personnel Mutual Aid Associations, Kumamoto Chuo Hospital, Kumamoto City, Kumamoto Japan; 4 Medical TOUKEI Corporation, Shinjuku-ku, Tokyo, Japan; 5 Medical Data Vision Co., Ltd., Chiyoda-ku, Tokyo, Japan; 6 Department of Corporate Planning, Linical Co., Ltd., Osaka City, Osaka Japan; 7 EU Biostatistics & Data Management, Daiichi Sankyo Europe GmbH, Munich, Germany; 8 Medical Science Department, Daiichi Sankyo Co., Ltd., Chuo-ku, Tokyo, Japan; 9 Safety and Risk Management Department, Daiichi Sankyo Co., Ltd., Chuo-ku, Tokyo, Japan; 10 Division of Biostatistics, Tohoku University Graduate School of Medicine, Sendai City, Miyagi Japan

**Keywords:** Venous thromboembolism, Pulmonary thromboembolism, Total knee arthroplasty, Total hip arthroplasty, Hip fracture surgery, Healthcare database

## Abstract

**Background:**

Orthopedic surgeries of lower extremities such as total knee arthroplasty (TKA), total hip arthroplasty (THA), and hip fracture surgery (HFS) are widely considered to carry a high risk of developing deep venous thrombosis (DVT) and pulmonary thromboembolism (PTE). Growing attention to epidemiological studies using a healthcare database led us to quantify the risks using a Japanese database to reveal recent medical care for such events.

**Methods:**

The study comprised 36,947 patients who had undergone orthopedic surgeries of the lower extremities and whose medical information from April 2008 to September 2013 was available. The source population of the database was derived from 100 acute-care hospitals with Diagnosis Procedure Combination. The events were defined by diagnosis, medication, imaging, and laboratory tests.

**Results:**

A breakdown of patients who underwent orthopedic surgeries by type of surgery showed 13.6% for TKA, 10.4% for THA, 56.8% for HFS, 1.5% for rupture of Achilles tendon, and 18.0% for simple fracture of lower extremities. The incidence for DVT, PTE, and bleeding events by type of surgery was 1.3, 0.2, and 1.0% for TKA; 0.9, 0.2, and 1.1% for THA; and 0.4, 0.1, and 1.8% for HFS, respectively. The population for risk factor analysis consisted of patients with similar background factors who underwent TKA, THA, or HFS. The statistically significant risk factors for PTE analyzed by the backward elimination procedure in a multivariate model were female sex, history of venous thromboembolism (VTE), thrombophilia, and varicose veins of lower extremity.

**Conclusions:**

The incidence of DVT, PTE, and bleeding events and the risk factors for DVT and PTE in patients by type of orthopedic surgeries of lower extremities found in our study are considered to be rational as they reflect evidence from real-world cases.

**Trial registration:**

University Hospital Medical Information Network Clinical Trials Registry UMIN000012667

**Electronic supplementary material:**

The online version of this article (doi:10.1186/s13018-017-0549-4) contains supplementary material, which is available to authorized users.

## Background

Venous thromboembolism (VTE) consists of deep venous thrombosis (DVT) and pulmonary thromboembolism (PTE). It has been evident that these diseases develop in relation to invasive orthopedic surgeries of the lower extremities, such as total knee arthroplasty (TKA), total hip arthroplasty (THA), and hip fracture surgery (HFS). To suppress the development of DVT and PTE in patients at high risk of VTE, sufficient anticoagulant therapy or intermittent pneumatic compression (IPC) in the early postoperative period is recommended in the Japanese guidelines [1, 2]. However, nationwide large-scale epidemiological data on VTE are still limited, so a true real-world understanding of the disease and treatment remains unclear.

In North America and Europe, electronic medical records are being used to examine real-world cases of the disease and treatment [3, 4]. In Japan, some databases of medical information have recently become available for this type of epidemiologic study. The launch of a healthcare database infrastructure project and the potential use of the Japanese National Database by the Ministry of Health, Labor, and Welfare could help to rapidly increase utilization of the healthcare database [5, 6]. Although the database in Japan still has limitations, such as the immaturity of methods for assurance and analytical procedures, it offers the advantages of enabling researchers to obtain the latest and hitherto unknown information more quickly compared with conventional prospective studies. A healthcare database is increasingly seen as an effective proposition for clinical studies.

Therefore, this study was conducted to examine the development, prophylaxis, or treatment of VTE and bleeding events using the healthcare database.

## Methods

### Data source

A hospital-based comprehensive database (EBM provider^®^) containing administrative data and laboratory values stored in hospital electronic information systems, which was constructed by Medical Data Vision Co., Ltd. (Tokyo, Japan), was used for this study. The source population of the database had aggregated approximately 9,000,000 patient records as of January 2015 and was derived from 167 acute medical care hospitals using the Diagnosis Procedure Combination (DPC) in Japan, which is 10% of all acute-care hospital inpatients across the country without regional bias.

EBM provider^®^ contains an anonymized patient identifier, along with sex, age, diagnoses, and complications, which were recorded in compliance with the International Classification of Disease, Tenth Revision (ICD-10) codes; operations, prescriptions, imaging, and laboratory values. EBM provider^®^ has been used effectively in multiple epidemiology studies [7, 8]. Thus, EBM provider^®^ was considered to be an appropriate medical database for patients who underwent an orthopedic operation of the lower extremities. However, the data cannot be traced to the source documents, because those documents are stored in a medical database separated from the personal information of patients and site information. Accordingly, there was a limitation in confirming the details of patient information when interpreting the study results and validation.

### Population

In this study, patients who underwent one or more of orthopedic operations of the lower extremities and who thus visited an acute-care hospital between April 2008 and September 2013 were identified and divided into five types of surgery (Table 1). The incident date of the orthopedic operation was determined by the computerized receipt code date corresponding to the effective date of the first lower limb orthopedic operation. The completion date of follow-up for each patient was the earlier of these dates: day 60 after the operation date, date of final record of the patient, or date of patient’s death.

**Table 1:** Classification of orthopedic surgeries of the lower extremities

Type of orthopedic surgery	Operation name of Japanese medical insurance claims procedure
Hip fracture surgery (HFS)	
	Open reduction and internal fixation (thigh)
	Open reduction of the intra-articular fracture (hip)
	Hemiarthroplasty (hip)
Total hip arthroplasty (THA)	
	Joint replacement surgery (hip)
	Joint re-replacement surgery (hip)
Total knee arthroplasty (TKA)	
	Joint replacement surgery (knee)
	Joint re-replacement surgery (knee)
Rupture of Achilles tendon	
	Achilles tendon rupture surgery
	Limb cast (half limb)
Simple fracture of lower extremities	
	Open reduction and internal fixation (lower leg)
	Open reduction and internal fixation (patella)
	Open reduction and internal fixation (foot)
	Open reduction of the intra-articular fracture (knee)
	Open reduction of the intra-articular fracture (foot)

### Sample size and power

The required sample size was estimated based on variation in the background rate of endpoints, magnitude of risk to be extracted, and exposure to risk factors to study the risk factor endpoints, with an α (significance level) of 0.05 (both sides) and a power of 80%. In a feasibility analysis, the number of patients who underwent orthopedic surgeries of the lower extremities (THA, TKA, and HFS) was 14,992 among the total of 2,289,670 patients from January 2011 to the end of June 2013. However, because the study period was extended for this study, the number of patients exceeded 15,000. When the endpoints with a 1% background rate were studied, it was possible to extract risk factors with a 10% exposure (control: exposure ratio of 9) and a risk ratio of 2 from a total of 15,120 cases.

### Statistical analysis

The incidence rate of VTE events and bleeding events (bleeding requiring blood transfusion, intracranial hemorrhage, intraocular hemorrhage, upper gastrointestinal bleeding, and lower gastrointestinal bleeding) were evaluated. VTE events and bleeding events were defined by combining ICD-10 codes; the definition of each event is in Additional file 1: Table S1. As recommended by the Guidelines for Epidemiological Studies for Safety Assessments of Medicines Using a Healthcare Database [9], before we conducted this study, we confirmed the validity of the criteria for VTE and bleeding events. We calculated the positive predictive value (PPV) based on the clinical laboratory data provided by the sites after the presence or absence of events was confirmed with laboratory data from a sample of medical records. The PPV is expressed as a probability of true events from detected events in the study. A total of 56 cases of VTE (39 cases of PTE, 17 of DVT) and 45 cases of bleeding events, all of which had laboratory data, were randomly selected and evaluated by two cardiologists and three orthopedists. The PPV was determined to be 75.0% for VTE, 76.9% for DVT, 70.6% for PTE, and 73.3% for any bleeding events, which indicates high reliability for identifying the incidence of VTE, DVT, PTE, and any bleeding events [10]. The methods for the data analysis for the present study and the definition of each event have been published elsewhere [11].

Logistic regression analysis was performed using 17 risk factors among those associated with VTE, including risk factors included in the guidelines [12] for the prevention of PTE and DVT. These risk factors were the history of VTE, thrombophilia, lower limb paralysis, lower limb casting, older age, protracted bed rest, congestive heart failure, respiratory failure, malignant disease, central venous catheterization, cancer chemotherapy, pregnancy, antipsychotic drugs, severe infections, obesity, estrogen replacement therapy, and varicose veins of the lower limbs. Protracted bed rest was excluded, because the database did not include such information.

The population for the risk factor analysis included only patients with similar background factors who underwent TKA, THA, or HFS. Exclusions were Achilles tendon rupture surgery (ATRS) and simple fracture surgery of the lower extremities (SFS).

A backward elimination procedure was used for the multivariate model (variable inclusion criteria; *P* = 0.05). Statistical analyses were performed using SAS software version 9.2 (SAS Inc. Cary, NC, USA).

## Results

### Patient background

From April 2008 to September 2013, outpatient and inpatient medical records of 5,044,743 patients were available in EBM provider. Of these patients, 36,947 who underwent orthopedic surgery of the lower extremities were identified. Table 2 shows the patient background by type of surgery. Specifically, 13.6% underwent TKA, 10.4% THA, 56.8% HFS, 1.5% ATRS, and 18.0% SFS. The mean age for HFS was the oldest, and 78.9% of them were older than 75 years. In contrast, the mean age was younger for ATRS and SFS. Regarding sex, the proportion of women was higher for TKA, THA, and HFS and lower for ATRS and SFS. The etiology at the time of surgery were osteoarthritis for TKA (81.2%) and THA (73.9%) and femoral neck fracture for HFS (88.2%). Mechanical prophylaxis was performed more frequently for TKA, THA, and HFS than for ATRS and SFS (Table 3).

**Table 2:** Patient demographics

Type of surgery	TKA	THA		HFS		Rupture of Achilles tendon		Simple fracture of lower extremities	
	5023 (13.6)^a^	3856 (10.4)^a^		20,991 (70.3)^a^		572 (1.5)^a^		6639 (18.0)^a^	
Sex									
Male	906 (18.0)	657	(17.0)	4815	(22.9)	385	(67.3)	3733	(56.2)
Female	4117 (82.0)	3199	(83.0)	16,176	(77.1)	187	(32.7)	2906	(43.8)
Age (years)									
<65	575 (11.4)	1518	(39.4)	1850	(8.8)	492	(86.0)	4302	(64.8)
65 to 75 years	1713 (34.1)	1173	(30.4)	2573	(12.3)	58	(10.1)	1237	(18.6)
≧75	2735 (54.4)	1165	(30.2)	16,568	(78.9)	22	(3.8)	1100	(16.6)
Mean ± SD	74.1 ± 7.9	67.4 ± 10.8		80.4 ± 12.7		45.5 ± 15.4		53.1 ± 21.4	
Body weight (kg)									
Mean ± SD	59.0 ± 11.1	55.6 ± 11.2		47.4 ± 10.4		67.6 ± 13.2		61.1 ± 13.7	
Body Mass Index (kg/m^2^)									
<18.5	93 (1.9)	246	(6.4)	4632	(22.1)	8	(14)	464	(7.0)
18.5 to 25.0	1878 (37.4)	2085	(54.1)	10,371	(49.4)	272	(47.6)	3543	(53.4)
≧25.0	2270 (45.2)	1133	(29.4)	1968	(9.4)	191	(33.4)	1594	(24.0)
Unknown	782 (15.6)	392	(10.2)	4020	(19.2)	101	(17.7)	1038	(15.6)
Mean ± SD	25.6 ± 4.0	23.6 ± 4.0		20.7 ± 3.6		24.5 ± 3.5		23.3 ± 4.2	
Etiology^b^									
Osteoarthritis	4078 (81.2)	2848	(73.9)	–		–		–	
Rheumatoid arthritis	252 (5.0)	82	(2.1)	–		–		–	
Idiopathic osteonecrosis	147 (2.9)	265	(6.9)	–		–		–	
Femoral neck fracture	–	–		18,518	(88.2)	–		–	
Others	573 (11.4)	687	(17.8)	2248	(12.1)	–		–	
Type of anesthesia									
Spinal anesthesia	293 (5.8)	56	(15)	8737	(41.6)	281	(49.1)	2760	(41.6)
Epidural anesthesia	2523 (50.2)	2318	(60.1)	1408	(6.7)	2	(0.3)	438	(6.6)
General anesthesia	2203 (43.9)	1480	(38.4)	10,768	(51.3)	189	(33.0)	3392	(51.1)

Abbreviations are the same as Table 1

^a^No. (%)

^b^There is some overlap

**Table 3:** Use of anticoagulant agents, antiplatelet agents, and mechanical prophylaxis

	TKA^a^	THA^a^	HFS^a^	Rupture of Achilles tendon^a^	Simple fracture of lower extremities^a^
	*n* = 5023	*n* = 3856	*n* = 20,991	*n* = 572	*n* = 6639
Anticoagulant agents					
Warfarin	436 (8.7)	219 (5.7)	1462 (7.0)	6 (1.0)	156 (2.3)
Unfractionated heparin	1369 (27.3)	1358 (35.2)	3574 (17.0)	3 (0.5)	397 (6.0)
Low molecular weight heparin	803 (16.0)	958 (24.8)	1003 (4.8)	0 (0.0)	47 (0.7)
Factor Xa inhibitor	3000 (59.7)	1937 (50.2)	3224 (15.4)	3 (0.5)	157 (2.4)
NOAC s	1443 (28.7)	816 (21.2)	1954 (9.3)	1 (0.2)	64 (1.0)
Fondaparinux	1570 (31.3)	1126 (29.2)	1280 (6.1)	2 (0.3)	93 (1.4)
Others	3 (0.1)	2 (0.1)	45 (0.2)	0 (0.0)	4 (0.1)
Antiplatelet agents	870 (17.6)	470 (12.2)	4645 (22.1)	17 (3.0)	513 (7.7)
Mechanical prophylaxis	4663 (92.8)	3583 (92.9)	18,191 (86.7)	308 (53.8)	4875 (73.4)

Other abbreviations are the same as Table 1

*NOAC s* novel oral anticoagulants (Edoxaban, Dabigatran, Rivaroxaban and Apixaban)

^a^No. (%)

Regarding anticoagulant agents, a factor Xa inhibitor was often used for TKA and THA, whereas it was less frequently used for HFS, ATRS, and SFS.

The incidence of PTE was very low and similar for TKA and THA (0.2%, respectively), while it was proportionally lower for HFS (0.1%) (Table 4). DVT was more common for TKA (1.3%) than for THA (0.9%) or HFS (0.4%). Only a few PTE events were observed in SFS, whereas the rate of DVT was similar with the rate for HFS. No PTE and VTE events were observed in ATRS.

**Table 4:** Venous thromboembolism (VTE) events incidence

Type of surgery		TKA			THA			HFS		Rupture of Achilles tendon			Simple fracture of lower extremities		
		*n* = 5023			*n* = 3856			*n* = 20,991		*n* = 572			*n* = 6639		
	*n*	%	95% CI	*n*	%	95% CI	*n*	%	95% CI	*n*	%	95% CI	*n*	%	95% CI
PTE events	8	0.2	0.0–0.3	6	0.2	0.0–0.3	29	0.1	0.1–0.2	0	–	–	3	0.0	0.0–0.1
DVT events	65	1.3	1.0–1.6	33	0.9	0.6–1.1	93	0.4	0.4–0.5	0	–	–	20	0.3	0.2–0.4
Any VTE events	68	1.4	1.0–1.7	36	0.9	0.6–1.2	102	0.5	0.4–0.6	0	–	–	20	0.3	0.2–0.4

Other abbreviations are the same as Table 1

*PTE* pulmonary thromboembolism, *DVT* deep venous thrombosis

The incidence of any bleeding events was 1.5%. Bleeding events generally were most frequently seen for HFS at 1.8%, while they were proportionally lower for THA (1.1%), TKA (1.0%), and SFS (1.1%). No bleeding complications were seen for ATRS (Table 5).

**Table 5:** Bleeding events incidence

Type of surgery	TKA			THA			HFS			Rupture of Achilles tendon			Simple fracture of lower extremities		
	*n* = 5023			*n* = 3856			*n* = 20,991			*n* = 572			*n* = 6639		
	*n*	%	95% CI	*n*	%	95% CI	*n*	%	95% CI	*n*	%	95% CI	*n*	%	95% CI
Bleeding requiring blood transfusion events	37	0.7	0.5–1.0	39	1.0	0.7–1.3	276	1.3	1.2–1.5	0	–	–	40	0.6	0.4–0.8
Intracranial bleeding events	1	0.0	0.0–0.1	2	0.1	0.0–0.1	36	0.2	0.1–0.2	0	–	–	17	0.3	0.1–0.4
Intraocular bleeding events	2	0.0	0.0–0.1	0	–	–	8	0.0	0.0–0.1	0	–	–	5	0.1	0.0–0.1
Upper gastrointestinal bleeding events	7	0.1	0.0–0.2	3	0.1	0.0–0.2	74	0.4	0.3–0.4	0	–	–	14	0.2	0.1–0.3
Lower gastrointestinal bleeding events	2	0.0	0.0–0.1	0	–	–	21	0.1	0.0–0.1	0	–	–	2	0.0	0.0–0.1
Any bleeding events	48	1.0	0.7–1.2	43	1.1	0.8–1.4	386	1.8	1.7–2.0	0	–	–	74	1.1	0.9–1.4

Abbreviations are the same as Table 1

The 60-day mortality rate by type of surgery was 0.1% (5 patients) for TKA, 0.2% (8 patients) for THA, 1.6% (337 patients) for HFS, 0.2% (12 patients) for SFS, and none for ATRS.

The significant risk factors in a multivariate model for DVT were female sex (odds ratio [OR], 1.9), history of VTE (OR, 11.4), thrombophilia (OR, 3.0), and older age (65 to 75 years, OR, 2.5; ≥75 years, OR, 2.1 vs <65 years). The significant risk factors for PTE were female sex (OR, 3.5), history of VTE (OR, 9.8), thrombophilia (OR, 4.7), and varicose veins of lower extremity (OR, 10.9) (Table 6).

**Table 6:** Univariate and multivariate odds ratio of PTE and DVT in patients who underwent TKA, THA, and HFS

	Total no. of patients	PTE no. (%)	PTE		DVT no. (%)	DVT	
Risk factors^†^			Univariate model	Multivariate model		Univariate model	Multivariate model
			Odds ratio (95% CI)	Odds ratio (95% CI)		Odds ratio (95% CI)	Odds ratio (95% CI)
Type of surgery							
HFS	20,986	29 (0.1)	1	–	93 (0.4)	1	1
TKA	5023	8 (0.2)	1.1 (0.5–2.5)	–	65 (1.3)	2.9 (2.1–4.0)***	2.7 (1.9–2.7)***
THA	3856	6 (0.2)	1.1 (0.5–2.7)	–	33 (0.9)	1.9 (1.3–2.9)**	2.1 (1.4–3.2)***
Sex							
Male	6378	3 (0.0)	1	1	22 (0.3)	1	1
Female	23,487	40 (0.2)	3.6 (1.1–11.7)*	3.5 (1.1–11.3)*	169 (0.7)	2.1 (1.3–3.3)**	1.9 (1.2–2.9)**
History of VTE							
No	29,816	42 (0.1)	1	1	187 (0.6)	1	1
Yes	49	1 (2.0)	14.8 (2.0–109.5)**	9.8 (1.2–77.5)*	4 (8.2)	14.1 (5.0–39.6)***	11.4 (4.0–32.3)***
Thrombophilia							
No	29,582	41 (0.1)	1	1	184 (0.6)	1	1
Yes	283	2 (0.7)	5.1 (1.2–21.3)*	4.7 (1.1–19.8)*	7 (2.5)	4.1 (1.9–8.7)^***^	3.0 (1.4–6.4)**
Elderly (age)							
<65	3941	5 (0.1)	1	–	14 (0.4)	1	1
65 to 75	5458	9 (0.2)	1.3 (0.4–3.9)	–	54 (1.0)	2.8 (1.6–5.1)***	2.5 (1.4–4.5)**
>75 year	20,466	29 (0.1)	1.1 (0.4–2.9)	–	123 (0.6)	1.7 (1.0–3.0)	2.1 (1.1–3.7)*
Antipsychotic drug							
No	26,958	42 (0.2)	1	–	182 (0.7)	1	–
Yes	2907	1 (0.0)	0.2 (0.0–1.6)	–	9 (0.3)	0.5 (0.2–0.9)^*^	–
Obesity (body mass index )							
<18.5	4971	4 (0.1)	1	–	20 (0.4)	1	–
18.5 to 25	14,331	20 (0.1)	1.7 (0.6–5.1)	–	87 (0.6)	1.5 (0.9–2.5)	–
≧25	5369	13 (0.2)	3.0 (1.0–9.3)	–	54 (1.0)	2.5 (1.5–4.2)***	–
Unknown	5194	6 (0.1)	1.4 (0.4–5.1)	–	30 (0.6)	1.4 (0.8–2.5)	–
Varicose veins of lower extremity							
No	29,767	41 (0.1)	1	1	189 (0.6)	1	–
Yes	98	2 (2.0)	15.1 (3.6–63.3)***	10.9 (2.5–47.5)**	2 (2.0)	3.3 (0.8–13.3)	–

Abbreviations are the same as Tables 1 and 4.

*p < 0.05; **p < 0.01; ***p < 0.001

^†^Risk factors with statistical significance are shown. The following factors without statistical significance are not listed: lower limbs paralysis, lower limbs casting, congestive heart failure, respiratory failure, malignant disease, central venous catheter, cancer chemotherapy, pregnancy, severe infections, and estrogen replacement therapy

## Discussion

Few large-scale epidemiologic data have been available concerning the incidence of DVT, PTE, and bleeding events as well as the risk factors for DVT and PTE in Japanese patients who experience orthopedic surgery of the lower extremities. The present study using the novel approach of a healthcare database has found in this patient population that the incidence of DVT, PTE, and bleeding events and the risk factors for DVT and PTE are similar to data obtained in previous studies. Thus, our present study data endorses the accuracy of the data collected through previously conventional methods.

We believe it is remarkable that our data allows us to confirm the accuracy of this approach of using the healthcare database and that this methodology holds promise for providing real-world insights into the management and treatment of disease and for predicting the prognosis. We anticipate that this also will be a useful tool to analyze the use of novel oral anticoagulants (NOACs); the rate of prescriptions for NOACs is expected to increase in Japan.

An advantage of the healthcare database is the sufficiently broad population to extract data on outcomes that have a low rate of occurrence. Yet, a limitation is the inability to confirm detailed patient data and interpret results, because the source data is not linked to the healthcare database. Thus, we conducted a validation study to verify the accuracy of the definitions and criteria used for the main analysis. This validation study yielded a high PPV for VTE events, and, as we previously reported [11], for DVT, PTE, and bleeding events. The negative predictive value was not assessed for feasibility; it is not clear whether the incidence might have been overestimated.

Two recent studies of TKA and THA conducted in Japan also provide insights into the patient characteristics and comparative data to our analysis. Specifically, the study using Japanese DPC data by Nagase and colleagues [13] and the Japanese multicenter cohort study reported by Migita and colleagues [14] found a similar distribution of patient characteristics as in our analysis. The ratio of TKA to THA was 1.4, 1.5, and 1.3 times; mean age was 69.9, 71.0, and 71.2 years; and female sex was 83.4, 83.9, and 82.0%, respectively. Regarding HFS, Orimo and colleagues [15] reported that the estimated incidence rates of new HFS patients in 2012 were 6.10/10,000 persons for men and 21.31/10,000 persons for women. The corresponding rates in our analysis were 9.54/10,000 persons for men and 32.07/10,000 persons for women. The ratio of women was similar with that in the research by Orimo and colleagues. Tsuda and colleagues [16] also reported similar results using Japanese DPC data.

The incidence of PTE (0.1%) in the present study was lower than that found by Nagase and colleagues (0.55%) and previously reported [13]. This difference can be explained by the differences in the criteria for each event. Our criteria tended to capture more apparent PTE and DVT cases, such as evidence of inferior vena cava filter placement or more than a month of anticoagulant agents treatment, in order to exclude false-positive cases involving the use of imaging only for checking. This means that the frequency found in the present study may be closer to that of the real world. Meanwhile, the incidence of PTE in this study was higher than the 0.06% rate reported by Kuroiwa and colleagues [17]. This difference could be the result of more patients with severe conditions undergoing anesthesiology. Additionally, the incidence of PTE was only 0.2% for TKA and none for THA in a study by Migita and colleagues [14]. Overall, the incidence of PTE in the Migita study matches the results in the present study.

In our study, symptomatic DVT events mostly occurred in TKA (1.3%), followed by THA (0.9%) and HFS (0.4%). This was similar with the report from Migita and colleagues of 0.9 and 0.2%, respectively. Tsuda and colleagues report a rate of 0.83% for symptomatic DVT events in the setting of HFS [16].

Among anticoagulant agents, factor Xa inhibitor was used most commonly, followed by unfractionated heparin. It is important to note that we used data for the period of April 2008 to September 2013, during which factor Xa inhibitors became available. Before that time, only unfractionated heparin or IPC was recommended for mechanical prophylaxis in the guidelines [18]. In Japan, insurance coverage for fondaparinux started in 2007, for enoxaparin in 2008, and for edoxaban in 2011. These agents are now recommended for anticoagulant in this setting.

Regarding the high bleeding risk for HFS in our study, a similar tendency was also observed in another study. In that study, the high risk of bleeding was associated with a low use of anticoagulants for HFS compared with TKA and THA. Orthopedic surgeons tend to use only mechanical prophylaxis for HFS, because of the high bleeding risk, and this is reflected in the reported results. However, given that the incidence of PTE and DVT was somewhat low, target patients for VTE prophylaxis should be chosen carefully depending on their risk factors for VTE and bleeding.

In this study, we identified that several risk factors for DVT and PTE were statistically significant: female sex, history of VTE, and thrombophilia. Although VTE risk factors have been stated in the Japanese guidelines, the evidence remains insufficient. Therefore, we believe the findings from this study provide valuable information to support evidence-based recommendations.

During follow-up, 362 patients died within 60 days in our study. Cushner and colleagues [19] reported a 90-day mortality of 0.1 to 0.3% in their TKA and THA patients. Thus, this suggests that the rate of 60-day mortality in our study for TKA and THA (0.1 to 0.2%) is feasible. Looking at HFS mortality, the 1.6% rate of mortality in our study appears to reflect the real-world clinical setting; the 30-day mortality rate for HFS of 7.5% reported by Khan and colleagues [20] and 5.3% by Neuman and colleagues [21] suggests mortality is higher for HFS than TKA or THA. The HFS population in all three studies was older. The high rate of mortality in the orthopedic field supports the importance of informed consent before surgery.

As with all studies, there were limitations that should be considered when interpreting the results of this study. The database used for this study had a broader definition of THA and TKA; hip resurfacing and bilateral THA were included in THA, whereas TKA included UKA, and the incidence of thrombotic or bleeding events for these may differ than those for simple THA or TKA. The database did not include the date that each event occurred; therefore, we determined this data based on the date of diagnostic imaging or other examinations and treatments related to the events. With this, whether the anticoagulant and antiplatelet was used for prophylaxis or treatment cannot be clearly distinguished, and these cannot be included in the risk factor analysis.

All analyses were conducted using only data that was within the electronic medical records, which excluded the assessment of the role of some risk factors. For example, creatinine clearance was not assessed because this was measured in only 16% of patients. Likewise, alcohol consumption and smoking was not assessed, as this was not recorded in the electronic medical record.

The lack of source data made it difficult to identify the cause of death. The 30-day rate of mortality for HFS reported in our study is less than one-third the value reported by the other studies discussed. This may reflect the different demographics in Japan or the ability to capture mortality data using this database. This may mean that this database is unable to identify fatal VTE cases among patients who undergo HFS. We also must note that because open reduction and internal fixation (thigh) are included in the definition of HFS, the study population includes some patients with femoral shaft fracture. However, given that almost 90% of HFS patients had a femoral neck fracture as the primary disease at surgery, the influence of contamination of some femoral shaft fractures on the estimate of VTE incidence and risk factor analysis should be limited.

## Conclusions

The limitations notwithstanding the overview of patient backgrounds, anticoagulant agents, and mechanical prophylaxis corresponded to those in the real world. As such, this study should be of value in various situations such as medical transition analysis and further risk factor analysis of VTE events. It should also help with the investigation of effects and side effects, using more accumulated data with larger populations for study parameters in the database in the future.

## Additional file

Additional file 1: Table S1. Definitions of events. (DOCX 18 kb)

## Abbreviations

ATRS: Achilles tendon rupture surgery
DPC: Diagnosis Procedure Combination
DVT: Deep venous thrombosis
HFS: Hip fracture surgery
ICD-10: International Classification of Disease, Tenth Revision
IPC: Intermittent pneumatic compression
NOACs: Novel oral anticoagulants
OR: Odds ratio
PPV: Positive predictive value
PTE: Pulmonary thromboembolism
SFS: Simple fracture surgery of the lower extremities
THA: Total hip arthroplasty
TKA: Total knee arthroplasty
VTE: Venous thromboembolism
