# Supplementary material for: Incidence of venous thromboembolism and bleeding events in patients with lower extremity orthopedic surgery: a retrospective analysis of a Japanese healthcare database
Source: J Orthop Surg Res. 2017 Apr 4;12:55. doi: 10.1186/s13018-017-0549-4 (PMC5381070; doi:10.1186/s13018-017-0549-4)
Supplement: Additional file 1: Table S1. — Definitions of events. (DOCX 18 kb) [file 13018_2017_549_MOESM1_ESM.docx]

Additional File , Table S1 　　　Definitions of events

|  | Event category | Event incidence criteria |
| --- | --- | --- |
| VTE events | PTE and DVT | Meets all the following criteria (1)-(5):  (1) Outpatient diagnosis of PTE/DVT, diagnosis on hospital admission indicated by a disease name code matching any one of those for PTE/DVT, or diagnosis of PTE/DVT during hospitalization (PTE: I26, DVT: I80, I82, O22, O87, O88), with recurrent PTE/DVT during hospitalization excluded to ensure one PTE/DVT episode per hospitalization  (2) Diagnostic imaging performed on the day of PTE/DVT onset  (3) Treatment for PTE/DVT^*1^ performed on the day of PTE/DVT onset or the following day  (4) Examinations related to PTE^*2^ performed within 7 days after PTE onset  (5) Either prolonged prescription of anticoagulants after PTE/DVT onset or death recorded within one month after the diagnosis of PTE/DVT |
| Bleeding events | Bleeding requiring transfusion | Meets the following criteria (1)-(4):  (1) No history of transfusion immediately prior to the day of orthopaedic surgery of the lower extremity or the first onset of VTE  (2) Transfusion of blood >800 mL after the day of orthopaedic surgery of the lower extremity or the first onset of VTE  (3) No surgery other than endoscopic hemostasis prior to or after the transfusion  (4) Transfusion on the day of surgery or the following day excluded |
|  | Intracranial bleeding | Meets the following criteria (1)-(3):  (1) MRI or CT examination performed after orthopaedic surgery of the lower extremity or the first onset of VTE (including within the same day)  (2) Any disease name code corresponding to a ICD-10 code for cerebrovascular diseases (I60-69) or intracranial injury (S06) recorded in the same month with the date of MRI or CT examination, with sequelae (I690, I691, I694) and chronic subdural hematoma (disease name code: 4321006, 4321008, 8842898) excluded  (3) Either rehabilitation for stroke or death recorded within 30 days after MRI or CT examination |
|  | Intraocular bleeding | Meets the following criteria (1) and (2):  (1) Ophthalmologic examination^*3^ performed after the day of orthopaedic surgery of the lower extremity or the first onset of VTE  (2) Any disease name code corresponding to any of the ICD-10 codes for Diseases of the eye and adnexa (H00-H59), Contusion of eyelid and periocular area (S00.1), and Contusion of eyeball and orbital tissues (S05.1) excluding Subconjunctival hemorrhage (H11.3) recorded in the same month with the date of the ophthalmologic examination |
|  | Upper gastrointestinal bleeding | Meets the following criteria (1)-(3):  (1) Examination related to upper gastrointestinal bleeding performed after the day of orthopaedic surgery of the lower extremity or the first onset of VTE  (2) Any disease name code corresponding to any of the ICD-10 codes for Diseases of oesophagus, stomach and duodenum (K20-K31) and Gastrointestinal hemorrhage, unspecified (K92.2) excluding those without hemorrhage or perforation (K253, K257, K259, K263, K267, K269, K277, K279, K287, K289) recorded in the same month with the date of the examination in (1)  (3) No anticoagulants prescribed on the day following the examination |
|  | Lower gastrointestinal bleeding | Meets the following criteria (1)-(3):  (1) Examination related to lower gastrointestinal bleeding performed after the day of orthopaedic surgery of the lower extremity or the first onset of VTE  (2) Any disease name code corresponding to any of the ICD-10 codes for Other diseases of intestines (K55-K63), Diseases of peritoneum (K65-K67), and Melaena (K92.1) recorded in the same month with the date of the examination in (1)  (3) No anticoagulants prescribed on the day following the examination |

*1: Treatment involving either prescription of heparin (>= 5,000 U) or Fondaparinux, IVC filter placement, or prescription of a thrombolytic agent (urokinase, t-PA products, etc.)

*2: Examination involving any of electrocardiography, radiography, echocardiography, oxygen administration, blood gas analysis, and respiration and heart rate monitoring

*3: Any of fundoscopy (unilateral), slit-lamp examination, fundus photography, 3-dimensional imaging of the fundus, or specular microscopy
